# A large scale statistical analysis of quantum and classical neural networks in the medical domain

**DOI:** 10.1038/s41598-025-33825-7

**Published:** 2026-01-09

**Authors:** Francesco Ghisoni, Matteo Borrotti, Paolo Mariani

**Affiliations:** 1 Physics department, Università degli Studi di Pavia, Via Agostino Bassi, 6, 27100 Pavia, Italy; 2 Department of Economics, Management and Statistics, University of Milano-Bicocca, Piazza dell’Ateneo, 1, 20126 Milan, Italy

**Keywords:** Quantum physics, Statistics

## Abstract

Classical neural networks (NNs) have shown strong performance in medical data analysis. However, they typically require large labeled datasets and may struggle in data-scarce scenarios, common in clinical practice. Quantum Neural Networks (QNNs) have emerged as a promising alternative. This paper presents a comparative study between NNs and QNNs for heart disease prediction, addressing the limitations of current models in low-data regimes. We systematically evaluate 460 QNNs (using 11-13 qubits) and 4,480 NN architectures, analyzing key design parameters: encoding schemes, re-uploading strategies, circuit depth, and dropout (for QNNs), as well as hidden layers, neurons per layer, and dropout (for classical NNs). Top-performing models are selected for a direct comparison in terms of accuracy and sample complexity. Our results show QNNs achieve comparable accuracy and demonstrate potential advantages in data-scarce settings. Our study presents a structured and reproducible methodology for evaluating QNNs in clinical contexts, thereby supporting the broader investigation of quantum machine learning in applied healthcare domains.

## Introduction

Machine Learning(ML), and in particular neural networks (NNs), is set to offer opportunities to improve patient health, support clinical teams with improved knowledge, reduce healthcare costs, and help deliver targeted healthcare ^1^. In fact, recent advances have demonstrated the successful application of classical neural networks and deep learning models across a wide range of medical contexts, including brain tumor detection from MRI scans using deep or shallow convolutional networks ^2,3^, automatic colorectal cancer classification from histopathological images ^4^, and telemonitoring systems for managing high-risk pregnancies ^5^. At the same time, the literature is seeing a growing interest in non-standard neural architectures, such as quaternion-valued echo state networks for speech emotion recognition ^6^. These studies highlight both the flexibility and the limitations of classical models in diverse medical settings.

An area of research that has grown in parallel with AI is quantum computing. With the first ideas dating back to the 1950s ^7^, this field seeks to change the computational paradigm. Just like their classical counterparts, quantum computers are universal machines ^8^ (i.e., any algorithm is computable both on a quantum and on a classical computer), with the key difference that some operations in quantum computing have no efficient classical counterpart. One key difference between classical and quantum computing is that, whereas the bit–the computational unit of a classical computer–can be either 0 or 1, the properties of quantum mechanics allow the qubit to be in any superposition of 0 and 1. This enables data processing in a way that is fundamentally different from classical computers. This new computing paradigm has enabled quantum algorithms that provide significant speed-ups for certain problems, impacting the fields of cryptography ^9^, simulation of quantum systems ^10^, and machine learning ^11^. In general, quantum algorithms are either polynomially or exponentially faster than their classical counterparts. The latter case includes important algorithms such as the Quantum Fourier Transform ^12^.

Over the last two decades, the construction of functional quantum computers has been addressed by both academia and industry, both of which have sought to build a fault-tolerant (error-resilient) quantum computer using different approaches such as superconducting qubits ^13^, photonic platforms ^14^, trapped ion technology ^15^, neutral atoms ^16^, and Nuclear Magnetic Resonance (NMR)^17^. The current era of available devices is known as the Noisy Intermediate Scale Quantum (NISQ) era^18^. Some devices are publicly accessible, but they have only a minimal number of noisy qubits, making it effectively impossible to implement known quantum algorithms that solve practical problems.

Despite hardware limitations, the research community has sought to leverage available quantum devices to solve valuable problems. One topic of significant interest has been Variational Quantum Algorithms (VQAs)^19^. These hybrid classical-quantum algorithms seek to divide the workload between a quantum and a classical computer. VQAs form a broad class of algorithms applicable to a variety of problems. One key application is in machine learning. VQAs can be trained on data to find an optimal set of parameters. When queried with unseen data from the same dataset, the quantum circuit can then produce the correct output. Consequently, VQAs have been explored as potential Quantum Neural Networks (QNNs)^20^.

VQAs, and more generally quantum machine learning (QML), have gained significant attention for their potential to outperform classical models in specific computational tasks. While early studies focused primarily on theoretical complexity arguments, more recent works have begun to investigate practical quantum advantage in concrete applications ^21^. For instance, QML models have shown promise in achieving performance gains in imaging tasks involving classical data, such as ghost imaging reconstruction using hybrid quantum-classical systems^22^, as well as in quantum-native tasks like critical sensing enhanced by quantum reinforcement learning^23^. These advances hint at how QML methods may offer not only theoretical benefits but also empirical advantages in structured and noisy environments.

In this work, we advance the application of QNNs in medical data analysis by conducting an extensive comparative study on heart disease prediction using the widely recognized Heart Disease dataset^24^, a benchmark dataset in the medical domain. We do this by focusing on real-world clinical data, particularly relevant in medicine, where datasets are often small and imbalanced due to the difficulty of collecting large-scale patient records. The main contribution of our study is the systematic evaluation of a broad spectrum of QNN architectures, exploring 46 different configurations changing hyperparameters such as quantum circuit depth, encoding schemes, circuit architecture, and the addition of dropout, with our experiments utilizing either 11 or 13 qubits. Furthermore, each configuration is tested 10 times with 10 different initializations to gain statistical relevance. The scale of this analysis is what distinguishes this study from prior works. To ensure a rigorous comparison, we benchmark QNNs against a large number of NNs, which have different numbers of layers, numbers of nodes per layer, and the addition of dropout. This comparison aims to quantify the actual benefits of QNNs over classical methods, particularly in low-data regimes, a common challenge in medical applications. Our findings contribute to the growing discourse on whether quantum computing can offer tangible advantages in data-limited environments. A key strength of our work is the efficient simulation and implementation of quantum circuits through state-of-the-art methods, allowing us to process real-world medical data effectively and overcome computational bottlenecks that typically limit QNN experiments. Finally, our study sets a benchmark for future research at the intersection of quantum computing and medicine, offering a structured methodology for evaluating QNNs in real-world healthcare applications.

This manuscript is organized as follows. In “Related works”, we overview related works comparing quantum and classical methods. In “Theoretical background”, we introduce the fundamentals of neural networks, quantum computing, and the Variational Quantum Classifier (VQC). “Results” presents the primary studies carried out in this manuscript. In “Discussion”, we discuss the main findings, implications, limitations, and future research directions. Finally, “Conclusions” concludes the work. For further material, including details on computational resources, dropout and barren plateau studies, and comparisons with ML methods, refer to the Supplementary Information.

### Related works

In recent years, several studies have aimed to compare QML techniques to ML both within and outside the medical setting. We refer to the following works ^25,26^ for general review papers on techniques from QML applied to the medical setting. Table 1 offers a summary of the presented literature.

We are particularly interested in the methods used in studies comparing QNNs or Variational Quantum Support Vector Machines (VQSVM)^27^ to classical methods. In the work conducted by Havenstein et al.^28^, the authors study two datasets: the UCI ML Breast Cancer Wisconsin (Diagnosis) dataset ^29^ and the UCI ML Wine dataset ^30^. For these, they compare both Kernel Quantum Support Vector Machines (KQSVM) and VQSVM to a classical Support Vector Machine (SVM) and analyze for accuracy on the test datasets. They test their quantum circuits on both simulators and real hardware. On the Breast Cancer Wisconsin dataset, they find that the VQSVM on the simulator has a final test accuracy of $95\%$. On the same dataset, the KQSVM on real hardware has an accuracy of $80\%$, while on the simulator the same architecture has an accuracy of $100\%$. The classical SVM has an accuracy of $85\%$. In contrast, on the Wine dataset, the overall accuracy of the VQSVM on hardware was $93.33\%$, the accuracy of the VQSVM on the simulator was $100\%$, and the accuracy of the classical SVM was $90\%$.

Landman et al.^31^ compare three neural network approaches: a classical neural network, a quantum-enhanced network using quantum computation for weight calculations, and a novel Quantum Orthogonal Neural Network (QONN). These models are evaluated on the Pneumonia-MNIST^32^ and Retina-MNIST ^33^ datasets for binary classification accuracy. For the Pneumonia-MNIST dataset, they use quantum models with up to 5 qubits, while for the Retina-MNIST dataset they use quantum models with up to 9 qubits. Both training and inference are performed using classical backpropagation, either through simulators or real quantum hardware. For the quantum-assisted neural networks on Pneumonia-MNIST, test accuracy ranges from $79\%$ to $87\%$. On Retina-MNIST, their accuracy ranges from $60\%$ to $80\%$. For the QONN on Pneumonia-MNIST, test accuracy ranges from $76\%$ to $83\%$, while on Retina-MNIST it ranges from $68\%$ to $80\%$. The classical neural networks perform comparably, with accuracy ranging from $80\%$ to $85\%$ on Pneumonia-MNIST and from $70\%$ to $80\%$ on Retina-MNIST.

In a study by Ullah et al. ^34^, the authors analyze clinical data collected from multiple public hospitals in Baja California over the first eight months of 2020 and compare the performance of Enhanced Quantum Support Vector Machines (E-QSVM) and Quantum Random Forests (QRF) to that of classical Support Vector Machines (SVM) and Random Forests (RF) in the classification of COVID-19 patients. The authors use a 10-qubit model and evaluate its performance on the test dataset. The test accuracies of the E-QSVM and QRF models are $78\%$ and $75\%$, respectively, while the classical SVM and RF models achieve $77\%$ and $73\%$, respectively.

Within particle physics, Cugini et al. ^35^ perform multi-class classification on jet stream data from the Large Hadron Collider. They restrict QNNs to architectures using between 2 and 5 qubits. They investigate the impact of training set size and feature count on model test accuracy. They run quantum circuits on both simulators and quantum hardware. They find that the best-performing quantum model achieves a test accuracy of $66\%$, compared to $67\%$ for the best classical model.

Abdulsalam et al.^36^ use the UCI Cleveland benchmark dataset^37^ for binary classification of heart disease. They test VQSVM, QNNs, VQC, and a hybrid architecture that integrates VQSVM, QNN, and VQC components. All experiments are carried out on simulators using 5 qubits and are evaluated for accuracy against a classical Support Vector Machine (SVM) and a deep neural network. They report test accuracies of $88.52\%$ for the VQSVM, $86.84\%$ for the QNN, $86.89\%$ for the VQC, and $90.16\%$ for the hybrid architecture. The classical SVM and the DNN both achieve test accuracies of $85.24\%$.

Sunkel et al. ^38^ use the COVID-CT-MD dataset to perform both multiclass and binary image classification. They further validate on Pneumonia-MNIST, OrganAMNIST, and MedMNIST datasets. They test VQCs as well as a Dressed VQC (D-VQC) using 4 or 8 qubits with a maximum depth of 4 layers. They test different circuit architectures and data encoding techniques in the quantum circuits. All experiments are carried out on simulators. The quantum and classical models perform roughly the same for both multiclass and binary classification, achieving test accuracies ranging from $55\%$ to $60\%$ for multiclass classification, and from $70\%$ to $75\%$ for binary classification. Explicit test accuracies are not reported for the other datasets.

Yu et al. ^39^ use early-onset colorectal cancer data to predict patient mortality. They compare the performance of VQSVM and classical SVM models as a function of feature count, training set size, and outcome ratio. Results show that the QSVM achieves an AUROC of $0.863\%$, outperforming the classical SVM, which achieves an AUROC of $0.723\%$.

Krunic et al. ^40^ compare Support Vector Machines (SVM) and Quantum Kernel Methods (QKM) for classifying electronic health records. They evaluate test accuracy and investigate the effect of feature count on performance. Results show that the classical model achieves approximately $57\%$ accuracy, while the best quantum model reaches around $55\%$ accuracy.

Omar et al.^41^ use Arabic Asthma Tweets^42^ and a broader Arabic tweets dataset ^43^ to perform sentiment analysis, comparing Random Forest (RF) and Quantum Support Vector Machine (QSVM) models. They find that the classical model slightly outperforms the quantum model in test accuracy, achieving $92.41\%$ compared to $92.05\%$.

**Table 1:** Comparison of selected quantum and classical ML papers in medical and scientific settings.

Literature	Dataset(s)	Qubit count	Real hardware	Quantum architectures	Test accuracy (quantum)	Classical architectures	Test accuracy (classical)
Havenstein et al. ^28^	Breast cancer Wisconsin dataset ^29^ Wine dataset ^30^	2	Yes	Kernel quantum support vector machine (KQSVM) Variational quantum support vector (VQSVM)	$\hbox {VQSVM}_{\text {Cancer}}$: 95% (sim) $\hbox {KQSVM}_{\text {Cancer}}$: 100% (sim) $\hbox {KQSVM}_{\text {Cancer}}$: 80% (hardware) $\hbox {VQSVM}_{\text {Wine}}$: 100% (sim) $\hbox {VQSVM}_{\text {Wine}}$: 93.33% (hardware)	SVM	$\hbox {SVM}_{\text {Cancer}}$:85% $\hbox {SVM}_{\text {Wine}}$:90%
Ullah et al. ^34^	COVID-19 patient data from hospital in Baja California	10	No	Enhanced-QSVM(E-QSVM) Quantum random forest(QRF)	E-QSVM: 78% QRF: 75%	SVM RF	SVM: 77% RF: 73%
Landman et al. ^31^	Pneumonia MNIST ^32^ Retina MNIST ^33^	5 9	Yes	Quantum orthogonal NN (QONN) Quantum-Assisted NN (QENN)	Best $\hbox {QANN}_{\text {Pneumonia}}$: $87\%$ Best $\hbox {QONN}_{\text {Pneumonia}}$: $83\%$ Best $\hbox {QANN}_{\text {Retina}}$: $80\%$ Best $\hbox {QONN}_{\text {Retina}}$: $80\%$	Neural network (NN)	Best $\hbox {NN}_{\text {Pneumonia}}$: $85\%$ Best $\hbox {NN}_{\text {Retina}}$: $80\%$
Cugini et al. ^35^	Jet data from LHC	2-5	Yes	Quntum Neural Networks (QNNs)	$66\%$	Deep NNs	$67\%$
Abdulsalam et al. ^36^	UCI Cleveland benchmark dataset ^37^	5	No	VQSVM QNN Variational Quantum classifier (VQC) Quantum Hybrid	VQSVM: $88.52\%$ QNN: $86.84\%$ VQC: $86.89\%$ Quantum hybrid: $90.16\%$	SVM Depp NN	SVM: $85.24\%$ Deep NN: $85.24\%$
Sunkel et al. ^38^	COVID-CT-MD	4, 8	No	Dressed VQC (D-VQC)	$\hbox {D-VQC}_\text {Binary}$: $70\%-75\%$ $\hbox {D-VQC}_\text {Multiclass}$: $55\%-60\%$	Deep NN	$\hbox {Deep NN}_\text {Binary}$: $70\%-75\%$ Deep $\hbox {NN}_\text {Multiclass}$: $55\%-60\%$
Yu et al. ^39^	Colorectal cancer mortality	3	No	VQSVM	AUROC: 0.863	SVM	AUROC: 0.723
Krunic et al. ^40^	Electronic health records	5	Yes	Quantum kernel methods	$\sim 55\%$	SVM	$\sim 57\%$
Omar et al. ^41^	Arabic asthma tweets	N/A	No	QSVM	$92.05\%$	RF	$92.41\%$
Our work	Heart disease dataset	11–13	No	QNNs	${86.84}\%$	Deep NNs	${86.67}\%$

Bowles et al. ^44^ recently proposed reframing benchmarking practices within the QML community. They highlight that benchmarking in QML is highly nontrivial, with factors such as dataset selection, methodologies, and positivity bias potentially limiting study effectiveness. They propose shifting the focus of comparative studies from simply outperforming classical machine learning models to rigorously evaluating QML models’ performance on specific tasks.

Finally, recent theoretical work by Caro et al. ^45^ demonstrates that QNNs can achieve robust performance even with limited data. This finding motivates studying the impact of training set size on QNN accuracy, which has important applications in the medical field.

As shown in Table 1, our study differs from the presented works in one or more of the following ways: we use a larger number of qubits; train the same model 10 times to ensure convergence and statistical robustness; employ modern training techniques for QNNs such as local cost functions, small-angle parameter initialization, and dropout; test a greater number of classical and quantum models; and evaluate accuracy as a function of training set size. One limitation of our study, compared to some of the presented works, is the lack of hardware implementation; this is further discussed in Section 4.

## Theoretical background

This section provides the foundational concepts necessary for comparing NNs and QNNs. We introduce the basics of NNs in “Classical neural networks”, covering key architectural components and learning mechanisms. Next, in “Quantum computing”, we present an overview of quantum computing, outlining the fundamental principles that underpin quantum algorithms and computational models. Finally, in “Variational quantum classifier”, we explore the VQC, explaining its structure and operation as a quantum analog to NNs.

### Classical neural networks

A classical neural network ^46^ is a computational model inspired by how biological nervous systems, such as the brain, process information. It consists of an extensive network of interconnected processing units called neurons, which interact to learn functions. These neurons are organized into layers, typically an input layer, one or more hidden layers, and an output layer.

The process begins with the input layer, which introduces data into the network. This data is then passed through the hidden layers, where the actual computation and feature extraction occur, before reaching the output layer, where the final result or system response is produced. In a standard feedforward neural network (NN), information flows in one direction without feedback loops. Each layer of neurons is connected to the next via weighted connections, where the weights (represented as real numbers) determine the strength of influence between neurons. The NN’s performance depends heavily on these weights, as they govern how inputs are transformed into outputs. Within each layer, the network first computes the weighted sum of inputs, then applies an activation function to the aggregated signal before passing it forward. Activation functions introduce non-linearity, enabling NNs to learn complex patterns. The Rectified Linear Unit (ReLU) is widely used due to its simplicity and effectiveness: it outputs zero for negative inputs and passes positive inputs unchanged. A variant, Leaky ReLU, addresses ReLU’s dying neuron issue by outputting a small, non-zero value (scaled by a parameter $\alpha$) for negative inputs while retaining ReLU’s behavior for positives. The sigmoid function compresses inputs to a range between 0 and 1, making it useful for binary classification (where outputs can be interpreted as probabilities). However, it suffers from vanishing gradients in deep networks, limiting its utility compared to ReLU. During training, weights are iteratively adjusted to minimize prediction error via backpropagation ^46^. This algorithm calculates the error between predicted and target outputs, then propagates it backward to update the weights. Optimization is typically performed using gradient descent, where the learning rate ($\alpha$) controls the step size of weight updates to ensure stable convergence. To prevent overfitting, a regularization term (e.g., L2 penalty controlled by $\lambda$) penalizes excessively large weights, promoting better generalization ^47^.

NN training has benefited from overparameterization, which refers to cases where the number of parameters significantly exceeds the number of training samples. In such cases, the optimization landscape becomes devoid of poor local minima while preserving global minima, producing favorable optimization properties. However, overparameterization can lead to overfitting, where models memorize noise rather than learning underlying patterns. Techniques like dropout ^48^ retain the benefits of overparameterization while preventing overfitting by randomly removing neurons during training iterations. This introduces beneficial noise that improves generalization.

### Quantum computing

We now focus on the basics of quantum computing to understand QNNs ^49^. The development of quantum computing tools begins with operations on the most straightforward quantum system: a single qubit. A qubit is a system that can either be off, represented as the vector $|0\rangle = (1,0)^T$, or on, described as the vector $|1\rangle =(0,1)^T$. The properties of quantum mechanics allow for the qubit to be in a superposition of $|0\rangle$ and $|1\rangle$, until it is measured. This is represented by the generic state $| \psi \rangle$, which is a mathematical vector of dimension $2^1 = 2$, where the power of 1 is due to the fact that it’s a single qubit system, and takes the form $|\psi \rangle = (a, b)^T = a|0\rangle + b|1\rangle$. This essentially means that, in general, the qubit can be both on and off simultaneously. When measured, the qubit will return to either $|0\rangle$ or $|1\rangle$, and the probability of these events is given by the parameters *a* and *b*, which are known as the amplitudes. In particular, the modulus squared of these values, i.e., $|a|^2$ or $|b|^2$, is the probability of seeing the state either off ($|0\rangle$) or on ($|1\rangle$). Since they are probabilities, they satisfy $|a|^2 + |b|^2 = 1$. An operation on the qubit is a procedure that wishes to modify the values of *a* and *b*. Operations on a qubit must preserve $|a|^2 + |b|^2 = 1$ and are therefore described by $2\times 2$ unitary matrices. Among these, some of the most important are the Pauli rotation matrices, $R_x(\theta ), R_y(\theta ), R_z(\theta )$, which are single qubit operations that change the state of the qubit by an amount that is parameterized by the angle $\theta$, and are defined by Eq. (1).

1 $$\begin{aligned} & R_x(\theta ) = \begin{pmatrix} \cos \left( \theta / 2\right) & -i\sin \left( \theta / 2\right) \\ -i\sin \left( \theta / 2\right) & \cos \left( \theta / 2\right) \\ \end{pmatrix}, \nonumber \\ & R_y(\theta ) = \begin{pmatrix} \cos \left( \theta / 2\right) & -\sin \left( \theta / 2\right) \\ \sin \left( \theta / 2\right) & \cos \left( \theta / 2\right) \\ \end{pmatrix}, \nonumber \\ & R_z(\theta ) = \begin{pmatrix} e^{-i\theta / 2} & 0 \\ 0 & e^{ i\theta / 2} \\ \end{pmatrix}. \end{aligned}$$

For example the application of the $R_y(\theta )$ rotation to a qubit in the $|0\rangle$ state produces the state $\cos \left( \theta / 2\right) {|{0}\rangle } + \sin \left( \theta / 2\right) {|{1}\rangle }$. These operations are critical since any single qubit operation can be represented as a successive application of $R_z(\delta )$ followed by $R_y(\gamma )$ and finally another $R_z(\beta )$ for some angles $\beta , \gamma , \delta$. Single qubit operations alone are insufficient for a universal quantum computer, i.e., we need more than single qubit operations to implement any algorithm.

We now turn our attention to 2 qubit states. These systems are described by a vector of dimension $2^2 = 4$, where the power of 2 is due to the fact we are looking at a two qubit system, and takes the form $|\psi \rangle = (a, b, c, d)^T = a|00\rangle + b|01\rangle + c|10\rangle + d|11\rangle$ with the condition $|a|^2 + |b|^2 + |c|^2 + |d|^2 = 1$. We can apply both single-qubit operations to the individual qubits or 2-qubit gates on this system. 2-qubit gates are $4\times 4$ unitary matrices, but we will only concentrate on the CNOT and CZ gates. These operations use a control and a target qubit. If the control qubit is in state ${|{0}\rangle }$, nothing happens to the target qubit. If the control qubit is in state ${|{1}\rangle }$, then the target qubit is subject to $R_x(\pi ) = X$ (where a global phase is ignored) gate in the case of the CNOT, and $R_z(\pi ) = Z$ gate in the case of the CZ gate. The matrix representation of these gates can be seen in Eq. (2).

2 $$\begin{aligned} & \text {CNOT} = \begin{pmatrix} 1 & 0 & 0 & 0 \\ 0 & 1 & 0 & 0 \\ 0 & 0 & 0 & 1 \\ 0 & 0 & 1 & 0 \end{pmatrix} \nonumber \\ & \text {CZ} = \begin{pmatrix} 1 & 0 & 0 & 0 \\ 0 & 1 & 0 & 0 \\ 0 & 0 & 1 & 0 \\ 0 & 0 & 0 & -1 \end{pmatrix} \end{aligned}$$

For example, applying the CNOT to the state ${|{10}\rangle }$ will produce the state ${|{11}\rangle }$. Similarly, applying the CZ to the state ${|{11}\rangle }$ will produce the state $-{|{11}\rangle }$.

In general, a quantum computer will have *n* qubits. An *n* qubit system describes a vector of dimension $2^n$, where the sum of the squares of the coefficients equals 1. Then, we see a quantum algorithm as a set of single-qubit and two-qubit gates applied to the *n* qubit system. A quantum algorithm aims to modify the amplitudes such that either (i) the probability that the correct outcome will be measured is increased on measurement or (ii) the answer is encoded within the amplitudes themselves. Most algorithms, such as Grover’s amplitude amplification or the Quantum Fourier Transform, store the result in the amplitudes. In these cases, extracting the result requires running the circuit multiple times to collect the necessary statistics—the number of circuits required to run is known as the shot number.

### Variational quantum classifier

**Fig. 1:**
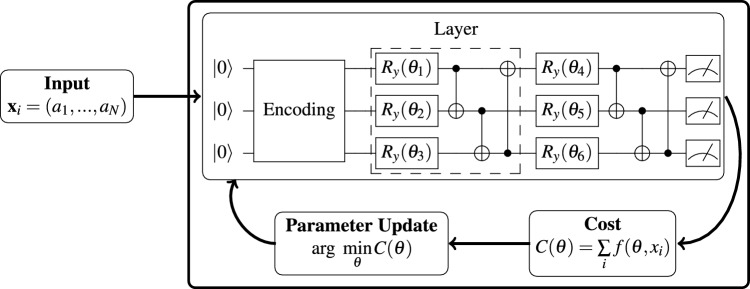
Schematic diagram of a variational quantum classifier. A data vector $\vec {x}$ is loaded on a quantum computer with a certain encoding technique. The data is then passed through a set of parameterized quantum gates acting as a neural network. A specific layer will combine 1 and 2 qubit gates, and multiple layers can be applied. At the end of the circuit, a measurement is performed, and the result is used to calculate a cost function, which is then used to update the parameters of the circuit. This training loop is performed on the entire dataset for multiple epochs until an ideal set of parameters can predict unseen data.

For the scope of this work, we present the Variational Quantum Classifier (VQC), a specific class of VQA that performs supervised binary classification ^50^. A diagram of a VQC can be seen in Fig. 1. This involves using a set of labeled data $(x_i, y_i)$ to train a quantum circuit such that when the circuit is fed unseen data $\tilde{x}$, it will output $\tilde{y} \in \{0,1\}$, which approximates the real solution *y*. On a foundational level, VQCs are grounded in works showing that, under the correct assumptions such as the Data Reuploading (DR) scheme, a VQC is a universal approximator ^51^.

Using the available data, VQCs are subjected to a training process that involves the repetition of the following 5 steps: (i) loading the data on the quantum computer, (ii) creating a quantum circuit that is dependent on a set of parameters $\vec {\theta }$, (iii) running the quantum circuit to get an output $\tilde{y}$, (iv) calculating a loss function that measures the distance between *y* and $\tilde{y}$, $\mathcal {L}(y,\tilde{y})$, and (v) backpropagating the loss to update the parameters.

While NNs benefit from abundant memory that easily handles binary data encoding, quantum computation faces fundamental limitations due to scarce qubit resources. This constraint necessitates specialized data loading techniques for VQCs, with the three most common approaches being basis (binary) encoding, amplitude encoding, and angle encoding. Mathematically, we represent the unitary that encodes the vector $\vec {x}$ onto a quantum state as $S(\vec {x})$. The importance of data loading cannot be overestimated since, without encoding techniques, it would be impossible to do any kind of QML.

Basis (binary) encoding is the quantum equivalent of binary encoding in the classical case. In particular, it is a type of encoding that can only be used with binary data. In particular, a *n*-dimensional binary string $\vec {x} \in (0,1)^n$ will be loaded onto *n* qubits, where the *i*th qubit will be in the state ${|{0}\rangle }$ or ${|{1}\rangle }$ depending on the value of the string element $x_i$. For example, the string 010 will be loaded onto 3 qubits, which will be in the state ${|{010}\rangle }$.

Amplitude encoding looks at encoding data in the coefficient of quantum states $|\psi \rangle$. For an *n* qubit system, the corresponding quantum state is a $N=2^n$ sized normalized vector with complex entries of the form $|\psi \rangle = (c_1, c_2,..., c_N)$, where $c_i$ are complex numbers. The normalized input vector $\vec {x}$ of size *N*, where some padding can ensure *N* is a power of 2, is loaded on $n=\log _2(N)$ qubits, and the data points are encoded in the coefficients $c_i$. This type of state preparation can be achieved through schemes such as Mottonen State Preparation, which requires on the order of $4^n$ single and 2-qubit gates ^52^. For example, loading a vector $\vec {x} = (0.4, 0.4, 0.8, 0.2)$ with amplitude encoding requires $\log _2(4)=2$ qubits and, after performing Mottonen state preparation, would produce the state $|\psi \rangle = 0.4|00 \rangle + 0.4|10 \rangle +0.8|01 \rangle +0.2|11 \rangle$.

Angle encoding encodes the data inside the rotation angles of Pauli rotations from Eq. (1). An input vector $\vec {x}$ of size *N* must first be pre-processed so that all the features are normalized in the interval $x_i \in [0, 2\pi ]$. Then, angle encoding will utilize *N* qubits and apply to each qubit a Pauli rotation (1) with angle $x_i$. For example, a vector with values $\vec {x} = (1, 0.75, 2.8, 4)$ will be encoded on 4 qubits, and the encoding technique will involve the application of a Pauli rotation, for example $R_y$ parametrized by the values of $\vec {x}$ ^53^.

The next critical aspect of VQCs is their ansatz, also known as parametric quantum circuit ^54^. To make an analogy, an ansatz is equivalent to the NN’s architecture. An ansatz is a quantum circuit composed of a combination of parameterized 1-qubit and 2-qubit gates and is denoted with the unitary $W(\vec {\theta })$. This combination constitutes a layer. A single VQC can have as many layers as needed. The specific structure of an ansatz typically depends on the task at hand, where factors such as input data are used to create an appropriate circuit. An essential property of an ansatz is its expressibility ^55^. This feature can generally be understood by how much of the entire space of possible quantum states the parameters $\vec {\theta }$ can explore. The ansatz for the VQC should be general enough general enough to guarantee the existence of an optimal set of parameters $\vec {\theta }^*$ that can give the correct output for the classification problem at hand. Therefore, a VQC will be composed of a parametric circuit that will include the application of the data loading unitary $S(\vec {x})$, followed by the ansatz $W(\vec {\theta })$. A particular way of casting multiple ansatzes is through the DR scheme. This scheme is analogous to ResNet ^56^, i.e., it performs the data uploading circuit every time a layer is repeated. So, the quantum circuit for a VQC with DR will start by encoding the data; this will be followed by a 1-layer of the ansatz, and then the data will be encoded again. This scheme ensures that VQCs can approximate any function and makes the optimization landscape easier to explore. We can write that the quantum circuit takes the form $\prod \limits _{l=1}^L S(\vec {x})W({\bf {\theta }})$.

Once an encoding technique and an ansatz are chosen, a quantum circuit is run, and the extraction of information from the quantum circuit requires a measurement. The measurement can be performed on all the qubits, which is known as a global measurement, or only on a subset of the qubits, which is known as a local measurement. For VQCs, the measurement has to be performed many times, i.e., using many shots, to gather some statistics. These statistics are then used to calculate the expectation values of some observable *O*. This is then used to calculate a loss function, which, for the VQC, is the Binary Cross Entropy (BCE) loss, Eq. (3).

3 $$\begin{aligned} \text {Binary Cross Entropy} = \frac{-1}{N} \sum _{i+1}^{n} y_i \log (p(y_i) + (1-y_i) \log (1-p(y_i)) \end{aligned}$$

It is important to note that local cost functions are much easier to train than global cost functions ^57^. Once the loss is calculated, the circuit parameters must be updated to minimize the loss function. This procedure can be done either with gradient-free optimization or gradient-based algorithms. In the latter case, backpropagation techniques are employed to update the parameters. These techniques require the calculation of gradients with respect to the input parameters. These can be easily calculated using the parameter-shift rule ^58,59^, a differentiation technique specific to quantum circuits. Once these have been computed, standard optimization techniques such as gradient descent can be used.

One major drawback in developing VQCs is the presence of barren plateaus in the training landscape of loss functions ^60^. This phenomenon results in the variance of the gradients being close to 0, which renders both gradient-based and gradient-free techniques for updating the parameters useless. QNNs that suffer from barren plateaus are deemed untrainable. A series of causes and solutions to barren plateaus have been identified ^61^, and we report two possible solutions. The first involves the utilization of a local cost function, in which the quantum circuits undergo only single-qubit measurements ^57^, while the second suggests the initialization of the parameters to small values ^62–64^.

### Quantum dropout

An analogue to classical dropout was recently introduced in work by Scala et al.^65^, which aims at reproducing the beneficial effects of dropout in NNs. Quantum dropout seeks to identify the single-qubit rotations in a VQC as the neurons and two-qubit gates as connections between neurons. In this view, quantum dropout becomes the random removal of single-qubit gates from the quantum circuit during the training phase to avoid phenomena such as overfitting or reliance on single parameters. The scheme has four steps: (i) the random selection of a QNN layer on which to perform dropout with probability $p_L$, (ii) the removal of single-qubit gates from the chosen layer with probability $p_G$, (iii) performing the optimization step for the circuit with the dropped operations, and (iv) iterating the procedure until termination of the optimization process. In general, $p_L$ and $p_G$ could be selected to increase the removal of specific layers/rotations. Yet, they can be considered uniform in the quantum setting since a VQC is composed of identical layer repetitions, unlike classical NNs. Therefore, we can write that the probability *p* of a specific rotation being dropped is $p = p_G p_L$. We note that despite the best setting to use dropout being in an overparameterized QNN, it has been shown that dropout can also be beneficial in training non-overparameterized networks^66^.

## Results

This section comprehensively analyzes QNNs and NNs for medical data classification. All the QNNs are implemented using Pennylane ^67^ through JAX ^68^ Just-In-Time (JIT) compilation and VMAP mapping to optimize training. Furthermore, Optax ^69^ is used for all QNNs to optimize the execution times maximally. All simulations were carried out on a high-performance compute cluster, which we estimated performed a total of 9,709 hours–approximately 404 days–of calculations and used a total of 15,947 GB of RAM. All details on the resources used can be found in the Supplementary Material. The simulations are carried out in the absence of shot and device noise. This choice is motivated by computational constraints: incorporating realistic noise models at the scale of this study, spanning hundreds of quantum neural network architectures, repeated training runs, and circuits involving up to 13 qubits would require density matrix simulations that are computationally infeasible, exceeding both memory and wall-clock limits of the available high-performance computing resources.

**Table 2:** Description of the features in the Heart Disease dataset.

Feature	Description
Age (Age)	Patient’s age in years, a significant CAD risk factor due to arterial aging
Sex (Sex)	Gender (1 = male, 0 = female), with males at generally higher risk
Chest pain type (ChestPainType)	Classification of chest pain: typical angina, atypical angina, non-anginal pain, or asymptomatic
Resting blood pressure (RestingBP)	Systolic blood pressure (mmHg) at rest, a critical hypertension-related variable
Serum cholesterol (Cholesterol)	Total serum cholesterol (mg/dL), associated with atherosclerosis risk
Fasting blood sugar (FastingBS)	Binary indicator of hyperglycemia (>120 mg/dL), associated with increased CAD risk
Resting ECG (RestingECG)	Electrocardiographic findings: Normal, ST-T wave abnormalities, or left ventricular hypertrophy
Max heart rate achieved (MaxHR)	Peak heart rate during a stress test, a marker of cardiac function
Exercise-induced angina (ExerciseAngina)	Chest pain triggered by exertion (1 = yes, 0 = no), indicative of coronary insufficiency
ST depression (Oldpeak)	Depression in the ST segment during exercise, a strong ischemia indicator
Slope of ST segment (ST_Slope)	Slope categories: Upsloping (normal), Flat (borderline abnormal), Downsloping (suggests ischemia)
Heart disease (HeartDisease)	Binary diagnosis of coronary artery disease (1 = present, 0 = absent)

### Dataset and pre-processing

The dataset used in this study is the *Heart Disease Dataset*, a benchmark in cardiovascular research, derived from a multi-center study on coronary artery disease (CAD) conducted by Detrano et al. ^24^. The dataset, compiled from patient records from institutions including the Cleveland Clinic, the Hungarian Institute of Cardiology, and the Veterans Administration Medical Center in Long Beach, California, provides valuable data to assess the likelihood of CAD through clinical and diagnostic parameters. CAD remains the leading cause of mortality worldwide, resulting in millions of deaths annually ^70^. The disease is due to the accumulation of atherosclerotic plaque in the coronary arteries, which reduces myocardial blood flow and leads to angina, dyspnea, myocardial infarction, or sudden cardiac death. Early detection is crucial to prevent complications and improve patient outcomes. However, a definitive diagnosis often relies on invasive procedures such as coronary angiography, which, although the gold standard, is costly and associated with procedural risks. Developing non-invasive diagnostic approaches using machine learning models trained on structured clinical data is a promising alternative, enabling rapid and accurate preliminary CAD assessments to prioritize patients for further testing.

The dataset comprises 914 patient records, each encompassing demographic, physiological, and biochemical attributes, along with the results of the electrocardiographic and stress tests. The binary target variable indicates the presence (1), 55% of the patients, or absence (0), 45% of the patients, of CAD. The dataset includes key features such as Age, a primary risk factor due to vascular aging; Sex, with males generally at higher risk, though postmenopausal females experience an increased likelihood of CAD; Type of chest pain, classified as Typical Angina, Atypical Angina, Non-Anginal Pain, or Asymptomatic, with typical angina strongly associated with CAD; Resting Blood Pressure, a critical hypertension-related variable; Serum Cholesterol, indicative of lipid metabolism and cardiovascular risk; and Fasting Blood Sugar, a binary indicator of hyperglycemia (>120 mg/dL), with diabetes significantly exacerbating the risk of CAD. In addition, resting ECG findings include regular readings, ST-T wave abnormalities suggesting ischemia, and left ventricular hypertrophy associated with chronic hypertension. The Max Heart Rate Achieved is a fitness marker, with lower values correlating with cardiac dysfunction. Exercise-Induced Angina, a marker of ischemic response during exertion, is highly indicative of compromised coronary circulation. ST depression during stress testing signifies myocardial ischemia, and the slope of the ST segment, classified as upward (normal), flat (borderline abnormal), or downward (highly suggestive of ischemia), enhances the diagnostic granularity. Table 2 summarizes these features.

This dataset includes features commonly utilized by cardiologists for CAD risk assessment and diagnostic decision-making. Machine learning models trained on structured patient data offer a promising solution to improve diagnostic accuracy and reduce reliance on invasive testing. By leveraging these features, AI-driven models can facilitate early disease detection, enhance risk stratification, and optimize patient management strategies. Given its diverse and well-structured feature set, this dataset provides a valuable foundation for evaluating the comparative effectiveness of classical and QNNs in medical decision support systems.

A preliminary data analysis assesses data distribution, detects anomalies, and identifies relevant patterns. No missing values are present in the dataset. Furthermore, no duplicate records are found.

**Fig. 2:**
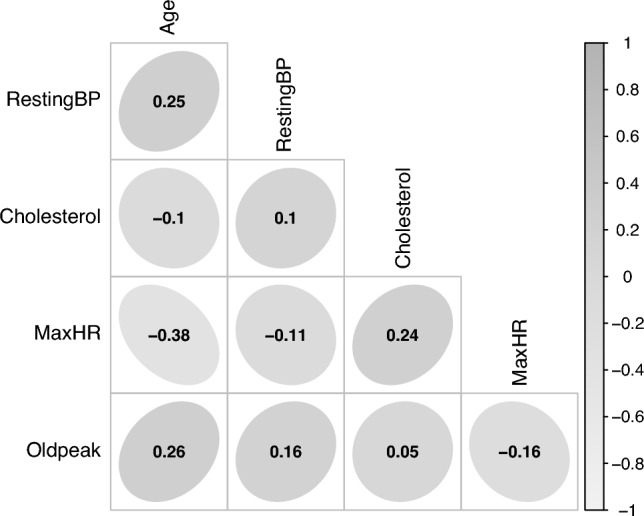
Correlation analysis.

Table 3 presents the descriptive statistics for the continuous variables. Figure 2 illustrates the correlation analysis among selected quantitative variables. Ellipses visually depict the direction and magnitude of correlations: elongated ellipses indicate stronger associations, whereas more circular shapes denote weaker relationships. Notably, Age shows a strong negative correlation with Maximum Heart Rate, consistent with established cardiovascular physiology. Additionally, a moderate positive correlation between Age and ST Depression (Oldpeak) highlights a potential increase in cardiac stress indicators among older individuals. Other variables, such as Resting Blood Pressure and Serum Cholesterol, reveals weaker or negligible linear correlations with the remaining variables.

**Table 3:** Summary statistics of continuous features.

Feature	Mean	Std dev	Min	Max	Median
Age	53.51	9.43	28	77	54
Resting BP	132.40	18.51	0	200	130
Serum cholesterol	198.80	109.38	0	603	223
Max heart rate	136.80	25.46	60	202	138
ST depression	0.89	1.07	-2.60	6.20	0.60

One-hot encoding is done for categorical variables such as Sex, Chest Pain Type, Fasting Blood Sugar, Resting ECG, Exercise-Induced Angina, and Slope of ST Segment. Various types of preprocessing are performed according to the different types of QNNs that are being trained. For QNNs that use amplitude encoding, non-categorical data is standardized according to the formula $z_i = (x_i - \mu _x)/\sigma _x$, where $\mu _x$ is the mean of column *x* and $\sigma _x$ is the standard deviation of the same variable. For the QNNs that use angle Encoding, all the data is shifted to be in the range [0,2$\pi$] using standard normalization techniques. The dependent variable is instead shifted from $\{0, 1\}$ to $\{-1, 1\}$ for all QNNs, to simplify the assignment when making a prediction. The dataset is subsequently randomly divided into training and test sets in a 70%-30% ratio, so the final datasets contain 643 and 275 observations, respectively.

### Experimental settings

The parameters optimized in our study for QNNs are the encoding technique, the number of layers, the ansatz repetition scheme, and the addition of dropout. Meanwhile, for NNs, we vary the architecture structure and the addition of dropout.

We tested both angle and amplitude encoding, as these are widely used techniques in the literature. The description of the preprocessing carried out on the data for both encoding techniques is reported in “Dataset and pre-processing”. When using angle encoding, we take a vector of data $\vec {x}$, and then, for each qubit, we apply a $R_y(x_i)$, where $x_i \in \vec {x}$, rotation. This means we use 11 qubits when using angle encoding. Instead, for the data loading process for amplitude encoding, we take the continuous variables, namely Age, Resting BP, Serum Cholesterol, Max Heart Rate, and ST depression, which have been standardized as indicated in “Dataset and pre-processing”, and encode them using Mottonen state preparation ^52^. Given these are 5 variables, the vector is padded with 3 zeros, and 3 qubits encode these values. The other data, i.e., the categorical values, are encoded using basis encoding. This means that, in total, the amplitude encoding circuit uses 13 qubits.

After the state preparation, the quantum circuit is subject to the ansatz. The ansatz we employ consists of a single layer of $R_y$ rotations on all qubits, followed by a “double structure” layer of CZ gates applied between pairs of qubits (e.g., qubits 1–2, 3–4, 5–6, etc.), a second layer of $R_y$ rotations excluding the first and last qubits, and a final “double structure” CZ layer also excluding the first and last qubits. The $R_y$ rotations use trainable parameters. This structured design balances expressibility and efficiency, making it suitable for 11- and 13-qubit implementations. Each layer *L* has a total of $(2N-1)L$ trainable parameters in the circuit, where *N* is the number of qubits. This is the ansatz that is used in all models. The minimum and maximum number of parameters can be seen in Table 5. While there is a significant discrepancy between the classical and quantum models, with the classical architecture reaching up to 17,655 parameters and the quantum one capped at 250, this disparity is intentional and reflects both practical and methodological considerations. On the one hand, the parameter limit of the QNNs arises from the constraints of current quantum simulators. On the other hand, classical models are deliberately allowed a much broader range of expressive power to ensure a stringent and unbiased benchmark. In particular, by training classical neural networks spanning from very low-capacity models to highly overparameterized architectures, we avoid artificially restricting classical performance. The number of layers varies between 1 and 10 for architectures without dropout, and 1-8 for architectures with dropout. The choices for the circuit structure and the number of layers are due to a trade-off between circuit size and the available computational resources. We employ simple ansatz repetition and the DR scheme to further enrich our study when using angle encoding. The latter has been shown to enhance the expressiveness of quantum circuits, enabling them to represent more complex decision boundaries. The DR scheme is not applied to amplitude encoding since this theoretically does not increase the expressiveness of the circuit but would highly increase the computational resources due to the circuit depth of Mottonen state preparation. A bias term is incorporated into all variational quantum classifiers, following the structure outlined in ^50^.

All experiments using dropout followed the scheme introduced by Scala et al. ^65^, where the layer dropout rate was set to $p_L = 0.1$, and the gate dropout rate was set to $p_G = 0.1$, for an overall dropout rate of $p=0.01$. This dropout rate was selected after we carried out an ablation study on the dropout rate and found 0.01 to be the optimal rate. Further details on the ablation study can be found in the Supplementary Material.

A local measurement is performed on the circuit. Precisely, the Pauli *Z* expectation value of each qubit is individually measured. Then, the sum of these expectation values is taken and passed through the sigmoid function to obtain an output. Local cost functions are preferred as they mitigate the barren plateau problem, which can hinder optimization. We use the Cross-Entropy loss as a loss function, Eq. (3).

In total, 46 different architectures are studied. Small-angle parameter initializations promote stable training where all the parameters are randomly sampled from a normal distribution with $\mu =1$ and $\sigma =1$ multiplied by 0.01. All experiments with QNNs use the Adam ^71^ optimizer with a variable learning rate, which starts at 0.01 and is multiplied by 0.5 if the cost function doesn’t decrease for 50 epochs. The optimization continues until the cost function doesn’t decrease for 100 epochs, which is when the training ends. This stabilizes the loss function to its minimum and avoids phenomena such as overfitting. In all experiments, we opt for 10 batches of size 64 during training to balance computational efficiency and generalization performance. A summary of all techniques and configurations tested is presented in Table 4.

**Table 4:** A table depicting all the tested variational quantum classifiers. The experiments involve modifying the encoding technique, the number of layers, the circuit architecture, and adding dropout.

Encoding	Architecture	Layers	Observables	Loss
Amplitude	Ansatz repetition	1–10	$Z \otimes I \otimes I \otimes \dots \otimes I$	
	Ansatz repetition + dropout	1–8	$I \otimes Z \otimes I \otimes \dots \otimes I$	
Angle	Ansatz repetition	1–10	.	Cross-entropy
	Ansatz repetition + dropout	1–8	.	
	Data-reuploading	1–10	.	
	Data-reuploading + dropout	1–8	$I \otimes I \otimes I \otimes \dots \otimes Z$	

Instead, for the NNs, we systematically vary two key hyperparameters: the number of hidden layers and the number of nodes per layer. The number of hidden layers ranged from 1–5, whilst the number of nodes per hidden layer ranged from 4–64. In order to have a manageable number of NNs to train without losing potentially performing models, we impose two main restrictions: only nodes that are multiples of 4 are tested, and every subsequent layer can have, at most, the number of nodes of the previous layer. This ensures that the total number of tested NNs, given that all configurations are tested with and without dropout, is 448. Each network uses the Leaky-ReLU with $\alpha = 0.1$ activation function in the hidden layers and the sigmoid activation function at the output layer. The binary cross-entropy loss function is employed to compute the error. To stabilize training, all networks with hidden layers use the He normal distribution to initialize weights and the Xavier initialization ^72^ to initialize biases. The perceptron, instead, uses the He uniform distribution ^56^ for weight initialization, and all biases are initially set to 0. The dropout rate is set to 0.2. The networks are trained using the Adam optimizer ^71^ with a variable learning rate, which starts at 0.001 and is multiplied by 0.5 if the cost function does not decrease for 100 epochs. All these techniques are chosen after initial experimental verification, which showed that they stabilized training for all networks. The training continues until the test cost function doesn’t decrease for 500 epochs, at which point it is stopped. This is done to ensure, just like with QNNs, the convergence of the optimization. To maintain consistency with the previous experiments on QNNs, the same batch size of 64 and number of batches are used across all trials.

**Table 5:** Minimum and maximum number of trainable parameters for each architecture considered in this study.

Architecture	Minimum number of parameters	Maximum number of parameters
QNN - Angle Encoding	25	250
QNN - Amplitude Encoding	21	210
Deep Neural Network	14	17,665

### Large-scale statistical analysis

In this section, we perform a comprehensive analysis of QNNs and NNs using accuracy on the testing dataset as the primary metric, which is especially relevant in medical data, where the stakes of incorrect predictions are high. This is also done to identify the optimal architecture for the study on sample complexity carried out in “Sample complexity study”.

**Fig. 3:**
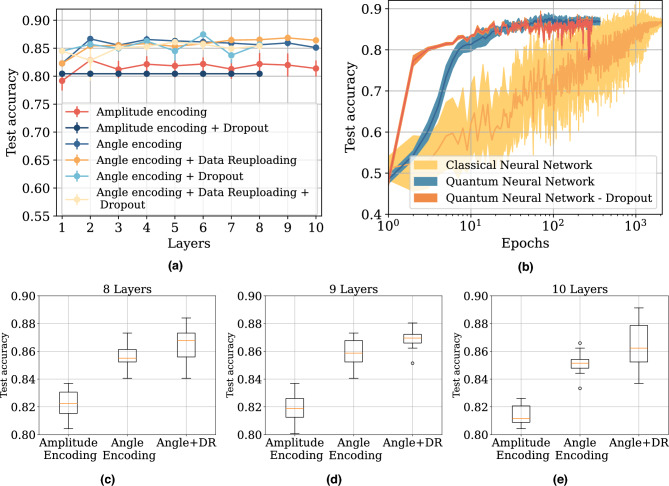
(**a**) Test accuracies for all QNNs as a function of the number of layers of the architecture. (**b**) The test accuracy as a function of the epochs for the best QNN with and without dropout, and NN. The overall accuracy is very close between the 3 networks, with the QNN with no dropout having a final test accuracy of 86.84%, the QNN with dropout having a final test accuracy of 87.46%, and the NN having a final test accuracy of 86.67%. (**c–e**) The box plots of the test accuracy for the 10 test runs for QNNs with amplitude, and angle encoding, also showing the results for angle encoding with data reuploading (DR).

The analysis is performed after testing all the proposed architectures in “Experimental settings”. One of the key elements of our study is that all the QNN and NN architectures are trained 10 times with 10 different random initializations to ensure statistical reliability. Therefore, we test 460 different QNNs and 4480 different NNs. In Fig. 3a, we show the results from all the tested QNNs, where we present test accuracy as a function of layers. In Fig. 3, we show test accuracy as a function of epochs for the best QNN and NN. All lines show both the average and the 95% confidence interval.

We start by looking at the results from the extensive experiments on QNNs shown in Fig. 3a. The results of the experiments suggest that angle encoding has a clear advantage over amplitude encoding. All configurations of amplitude encoding converge to a test accuracy between 80%–82%, whilst all configurations for angle encoding converge at a final test accuracy between 83%–87%. It is worth noting that this pattern is independent of the other techniques. Angle encoding, with two or more layers, always seems to bring an advantage in the training of classical data.

We now look at the impact of the number of layers on the final test accuracies for the QNNs with amplitude encoding. Amplitude encoding without dropout increases performance from 1 to 2 layers, with a subsequent plateau. The plateau accuracy occurs around a test accuracy of 82%. The confidence interval for the accuracy of amplitude encoding is quite limited, except in the case of 8 and 9 layers, where it becomes significant, suggesting an unstable learning process. This reflects considerable variability in the training outcomes, a highly undesirable characteristic for a QNN. Instead, for QNNs with amplitude encoding and dropout, the results suggest that the dropout rate takes over, and there is no significant increase in the accuracy as a function of the layers.

We now turn our attention to the results for angle encoding. Similarly, we also find an increase in the test accuracy from 1 to 2 layers, with a subsequent plateau. As mentioned, the plateau is higher in this case, staying around a test accuracy of 86%. In this case, the DR scheme performs slightly worse when compared to the ansatz repetition for less than 6 layers, with a subsequent takeoff. In both cases, the test accuracies for both architectures are with 2% test accuracy of each other for all layers. We note that the training is stable for all these layers, as indicated by the small error bars in the graphs. As mentioned before, this is desirable since the convergence is stable. Instead, for the QNNs with angle encoding and dropout, we find that the inclusion of dropout does not consistently improve performance. In fact, the test accuracy tends to fluctuate around the performance of the corresponding QNN without dropout. While dropout occasionally yields better results, it also often performs worse, suggesting that its effect is somewhat unstable. This variability is likely due to the additional noise introduced by dropout, which may not consistently benefit training in this regime. Overall, the two best-performing QNNs were: one with 9 layers, angle encoding, and DR, achieving a final test accuracy of 86.84%; and another without DR but with dropout, achieving a slightly higher final test accuracy of 87.46%. However, due to the observed instability and fluctuating performance of the latter, we chose the former as the reference model for further studies. In Fig. 3c, 3d, and  3ewe show box plots plotted using the test accuracies for 10 instances for QNNs with amplitude encoding, angle Encoding and angle Encoding with DR for QNNs with 8, 9, and 10 layers. The choice to show these settings is motivated by including the best QNN, the 9-layer design with angle encoding and DR, and highlighting the performance of QNNs close to this architecture in terms of layers and hyperparameters.

A similar study was conducted for the NNs with the experimental setup described in “Experimental settings”. We mainly state that the studies show the best-performing model to be an NN with three hidden layers with 52 nodes in each layer and no dropout.

In the direct comparison between the NN and the QNN shown in Fig. 3, we first focus on their overall accuracy. One primary observation is that the QNNs, with and without dropout, converge much faster than the NN during training, suggesting a more straightforward optimization landscape. We highlight that this is mainly due to the different initial learning rates of the two networks, with the QNN starting at 0.01 and the NN at 0.001. These values were chosen since initial experiments showed that a higher learning rate for the NN would hinder its convergence. However, despite this difference in convergence speed, all models ultimately achieve comparable final accuracies of 86.84%, 87.46%, 86.67% for the QNN with and without dropout, and the NN, respectively. A table summarizing the results can be found in Table 6.

While it is known that deep QNNs can, in principle, suffer from the barren plateau phenomenon—where gradients vanish exponentially with system size—we do not observe this issue in our study. In particular, we find that the gradients plateau at a value of 0.0167, which is high enough to allow for gradient descent methods to be applied. An in-depth analysis for the barren plateau issue can be found in the Supplementary Material. We emphasize that this observation is limited to the noiseless simulation regime considered in this work; the presence of noise may induce or exacerbate barren plateaus even in shallow circuits, an effect that cannot be assessed here due to the computational infeasibility of large-scale noisy simulations.

**Table 6:** This table provides a summarization of the results from our large-scale statistical analysis. For each architecture, we report only the best observed test accuracy across all experimental runs.

Architecture	Best test accuracy
QNN - amplitude encoding	82.86 ± 0.01%
QNN - amplitude encoding + dropout	80.43± 0.01%
QNN - angle encoding	86.67 ± 0.01%
QNN - angle encoding + data re-uploading	86.84 ± 0.01 %
**QNN - angle encoding + dropout**	**87.46** ± **0.01%**
QNN - angle encoding + data re-uploading + dropout	86.09 ± 0.01%
Classical neural network(52–52–52)	86.67± 0.07%

**Fig. 4:**
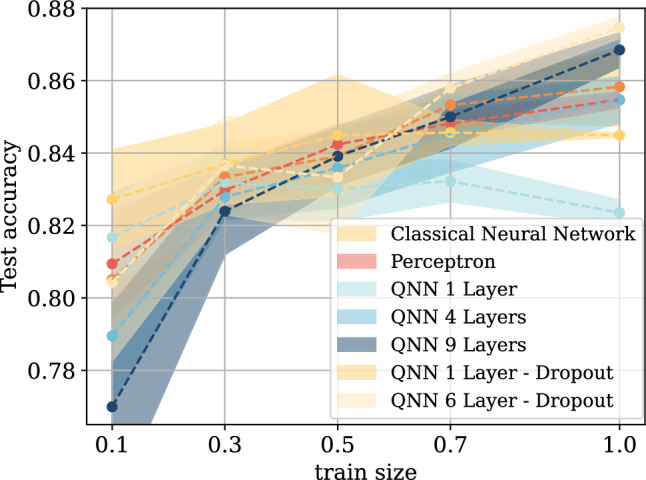
Results of the sample complexity study, showcasing how the performance of the networks varies with different training sample sizes.

### Sample complexity study

Having explored and tested a wide range of NNs and QNNs, and using the test accuracy as the metric for comparison, we now study the effect of the training dataset size, also known as sample complexity, on the test accuracy. Sample complexity becomes a vital aspect to consider because it reflects the efficiency with which a model learns from limited data. In many real-world scenarios, particularly in the medical field, obtaining large datasets can be costly or complicated, so a model’s ability to generalize well from fewer examples is essential.

To perform this study, we create subsets of the training data corresponding to 10%, 30%, 50%, 70%, and 100% of the original training set. For each subset size, we train QNNs with angle encoding, dropout, and either 1 or 6 layers. The choice of angle encoding with dropout is motivated by the fact that the initial experiments suggest these to be the most valid architectures, while several layers were varied to account for the varying complexity of the dataset. QNNs without dropout were also included to use as a benchmark, For the QNN without dropout we adopt the DR architecture since this proved to be the most performing architecture without dropout. For the NN, we trained both the best-performing architecture based on three hidden layers with 52 neurons each, as well as a perceptron, corresponding to a linear classifier. This choice was made to explicitly span the full spectrum of classical model complexity, from minimal-capacity models with low risk of overfitting to highly expressive architectures. Including both extremes ensures that the comparison with QNNs in the sample complexity study is not biased toward overly complex classical models. As a result, the observed performance of shallow QNNs in low-data regimes cannot be solely attributed to reduced model capacity, since equally simple classical baselines are explicitly considered. This latter choice was also made to account for the varying complexity of the dataset. All the networks are trained 10 times on a specific dataset size, using a different random sample of the subset to ensure statistical validity. In addition, different parameter initialization is used for each experiment. The results are reported in Fig. 4. A table summarizing the results can be found in Table 7.

From the results shown in Fig. 4, we observe that the single-layer QNN with dropout outperforms both the classical neural network (NN) and the perceptron when trained on small portions of the dataset, specifically at the smaller dataset sizes 10% to 50%. The second best performing architecture, for the smallest dataset size, is QNN with 1 layer. This behavior is consistent with recent theoretical findings suggesting that QNNs can generalize well with limited data availability ^45^, an especially relevant feature in medical applications where acquiring labeled data is costly and time-consuming. We note that the addition of dropout appears to be significantly helping the quantum networks, which we believe is due to the added noise facilitating the training landscape. As the training set increases, the performance of the single-layer QNN tends to plateau, both with and without dropout, while the classical models–particularly the NN–show a more substantial improvement, eventually surpassing the shallow quantum model. Interestingly, QNNs with dropout and 6 layers and without dropout with 4 and 9 layers display a different trend: their performance increases more markedly with training size, particularly between 10% and 30%, progressively narrowing the gap with the classical NN and continuing to improve with additional data. These observations suggest a trade-off in architectural choice: for low-data regimes, a 1-layer QNN is preferable due to its strong generalization from few samples; however, in scenarios with moderate data availability, deeper QNNs (4 or 9 layers) become more competitive and may be the better choice for practical use.

**Table 7:** Results from the sample complexity experiments. We report the test accuracy for all studied architectures at all training set sizes: 10% (0.1), 30% (0.3), 50% (0.5), 70% (0.7) and 100% (1.0) of the full dataset.

Architecture	0.1 train size	0.3 train size	0.5 train size	0.7 train size	1.0 train size
Perceptron	80.94 ± 0.02%	82.97 ± 0.01 %	84.24 ± 0.01%	84.84 ± 0.01 %	85.47 ± 0.01%
Classical neural network	80.51 ± 0.03%	83.33 ± 0.01 %	83.91 ± 0.03%	85.32 ± 0.01 %	85.83 ± 0.02%
QNN 1 layer	81.67 ± 0.02%	83.19 ± 0.02 %	83.00 ± 0.01%	83.22 ± 0.01 %	82.35 ± 0.01%
QNN 4 layers	78.94 ± 0.03%	82.79 ± 0.01 %	83.59 ± 0.02%	84.53 ± 0.02 %	85.47 ± 0.01%
QNN 9 layers	76.99 ± 0.05%	82.39 ± 0.02 %	83.91 ± 0.01%	85.00 ± 0.01 %	86.84 ± 0.01%
QNN 1 layer - dropout	**82.72 ± 0.02%**	**83.70 ± 0.02** %	**84.49 ± 0.03%**	84.57 ± 0.01%	84.49± 0.01%
QNN 6 layer - dropout	80.43 ± 0.04%	83.66 ± 0.02%	83.33 ± 0.03%	**85.80 ± 0.01%**	**87.46 ± 0.01%**

## Discussion

This study provides a systematic comparison between QNNs and NNs in the context of medical data classification, using the Heart Disease Dataset^24^. Our primary goal was to evaluate the two approaches based on accuracy and sample complexity. To this end, we tested 46 QNNs and 448 NNs, varying key parameters such as encoding techniques, number of layers, circuit architecture, and addition of dropout for the QNNs while systematically altering the number of hidden layers, nodes per layer, and addition of dropout for the NNs. Each configuration is tested 10 times to achieve statistical reliability, meaning a total of 460 QNNs and 4,480 NNs are tested as part of this study. After identifying the best-performing models in both categories, we compared their ability to learn from different training set sizes and evaluated their convergence speed.

Our findings show that QNNs generally converge faster during training and exhibit a more stable behavior and maintain competitive accuracy, particularly in low-data regimes. The optimal QNN configuration employed angle encoding, implemented the dropout scheme, and is based on 6 layers. Furthermore, we can see that while increasing the number of layers can improve results, there is a threshold beyond which additional layers yield diminishing returns. We take this QNN and compare it to the best-performing NN, with 3 hidden layers with 52 nodes per layer. Finally, we examine the sample complexity of the quantum and classical models, revealing that shallow QNNs with dropout outperform complex and classical models when trained on fewer data points. This result provides experimental evidence supporting a recent finding that QNNs may be able to generalize with fewer data points ^45^.

Compared to existing studies focusing on deep learning in medicine^3,4^, our work highlights the viability of QNNs in cases where data acquisition is expensive or constrained. Moreover, unlike most prior works that focus on classical architectures, this study provides one of the few large-scale empirical evaluations of QNNs for structured medical data, thereby filling a gap in the literature.

### Implications

From a clinical perspective, QNNs may serve as effective models for decision-support systems where limited training data is available, such as in rare disease diagnosis or low-resource settings. From a research standpoint, our work offers a reproducible framework to assess quantum models side by side with classical ones, facilitating future benchmarking as quantum hardware becomes more accessible. Additionally, our results suggest that the often assumed dominance of classical NNs may not hold in all settings, especially when training data are scarce, opening the door to new quantum-enhanced workflows for medical diagnostics.

### Limitations

We are aware that this study has some limitations that must be acknowledged. First, the analysis is restricted to a single, structured tabular dataset focused on binary classification. While this allows for controlled experimentation and direct comparison between architectures, it does not capture the broader diversity of medical data encountered in real-world settings.

Second, we did not explore other data modalities such as medical imaging, clinical text, or physiological time series, which are increasingly important in healthcare applications. These data types often require specialized processing pipelines and may involve significantly different model architectures. Although adapting QNNs to such domains is of great interest, it remains an open and technically challenging area of research, particularly due to constraints in data encoding, circuit depth, and quantum memory.

Third, our experiments were conducted on quantum simulators rather than real quantum hardware. While our results provide a useful benchmark for QNN performance, the absence of real hardware execution limits the generalizability of our findings. In particular, gradient calculations are significantly simplified in simulation due to the use of automatic differentiation, and do not suffer from shot noise or device-induced noise. Furthermore, the presence of noise can induce or exacerbate barren plateaus even in shallow circuits, an effect that cannot be assessed in our study due to the lack of noisy simulations. Additionally, the evaluation of quantum circuits in simulation is noiseless and deterministic, which can mask the challenges of barren plateaus or instability in real-world scenarios. Consequently, while simulators enable systematic and scalable experiments, they may overestimate the practical applicability and robustness of QNN architectures in near-term quantum devices. Nevertheless, conducting experiments in simulation remains a valid and essential step in the development of quantum machine learning methods. It enables controlled and reproducible benchmarking across different architectures, facilitates the design and debugging of novel training strategies, and allows researchers to study scaling behavior and performance trends that would be infeasible to explore on current hardware due to noise, limited qubit counts, or runtime constraints.

### Future work

Possible extensions to this study could include utilizing more computing power to test for deeper networks and reach the over parametrized regime. Additionally, further techniques for QNNs, such as dynamic encoding methods, should be tested, and dynamic ansatzes that may enhance model performance should be explored. Furthermore, future studies could expand the comparison between overall accuracy and sample complexity to multi-classification tasks, maintaining the focus on medical applications. This would provide a broader understanding of how these models perform in more complex scenarios, thereby informing their practical use in clinical settings where multi-class classifications, such as diagnosing multiple diseases, are often necessary.

Another important direction for future research involves extending the current analysis to unstructured data. These data types are increasingly prevalent in real-world healthcare scenarios and offer richer, more complex representations of patient states. Investigating the applicability of QNNs to such domains would require addressing significant challenges in different aspects of quantum computing. Despite these difficulties, exploring QNNs in unstructured settings is essential for assessing their broader potential and identifying use cases where quantum models may provide unique benefits over classical approaches. This line of research could also uncover novel quantum architectures or hybrid strategies tailored explicitly for visual or sequential medical data.

Furthermore, an important research area involves enhancing the interpretability of QNNs, particularly in high-stakes applications such as medical decision-making. Recent literature has highlighted the necessity of explainable models in healthcare, where transparency and trust are essential. While classical neural networks benefit from a wide range of interpretability tools, such as sensitivity analysis and surrogate models^73,74^, recent advances suggest that similar principles can be extended to QNNs. For example, local approximants and sensitivity-based approaches have been proposed to provide insight into QNN behavior^75^. Additionally, techniques like conformal prediction and leave-one-co variate-out (LOCO) analysis, which have shown promise in classical settings for assessing uncertainty and feature relevance, may also be adapted to the quantum domain in future work^76^. Integrating these methods into the QNN framework could significantly strengthen the transparency and accountability of quantum models in clinical environments.

## Conclusions

This work highlights the potential of QNNs for medical data classification in data-scarce scenarios, a context frequently encountered in clinical applications. Through a large-scale, systematic comparison with classical neural networks, we establish a replicable and structured framework for assessing quantum models in realistic healthcare settings. Rather than focusing solely on performance metrics, our findings emphasize that QNNs, despite current hardware limitations, can already offer competitive and, in some cases, advantageous behavior in structured data tasks. In particular, their stability and sample efficiency make them promising candidates for applications where data collection is constrained or costly. We believe that this study lays a solid foundation for the future integration of QNNs into medical workflows. It encourages further investigation into quantum model interpretability, implementation on quantum hardware, and the extension of quantum approaches to unstructured clinical data such as images, signals, and text. As quantum technologies mature, such directions will be essential to unlocking their full potential in healthcare and beyond.

## Supplementary Information

Supplementary Information.

## Data Availability

All the code and data used through the work can be found at the following at the following github link : https://github.com/fghisoni/Comparative-Analysis-of-Classical-and-Quantum-Neural-Networks-for-the-Classification-of-Medical-Data
