# Supplementary material for: A large scale statistical analysis of quantum and classical neural networks in the medical domain
Source: Sci Rep. 2026 Jan 9;16:3719. doi: 10.1038/s41598-025-33825-7 (PMC12852685; doi:10.1038/s41598-025-33825-7)
Supplement: Supplementary file 1 — Supplementary Information. [file 41598_2025_33825_MOESM1_ESM.pdf]

Supplementary Information: "A Large Scale Statistical Analysis of Quantum and Classical Neural Networks in the Medical Domain"

Francesco Ghisoni,[1,*] Matteo Borrotti,[2] and Paolo Mariani[2]

[1] *Physics department, Università degli Studi di Pavia, Via Agostino Bassi, 6, Pavia, 27100, Italy*
[2] *Department of Economics, Managment and Statistics, University of Milano-Bicocca, Piazza dell'Ateneo, 1, Milano, 20126, Italy*

(Dated: December 17, 2025)

I. COMPUTATIONAL RESOURCES

In Table I and Table II, we report the simulation time and peak memory usage for each individual QNN architecture tested. The first column of each table lists the different quantum architectures considered in this study: Angle Encoding, Angle Encoding + Data Reuploading, Angle Encoding + Data Reuploading + Dropout, Amplitude Encoding, and Amplitude Encoding + Dropout. The remaining columns correspond to the number of ansatz layers used in each architecture—ranging from 1 to 10 for architectures without dropout, and from 1 to 8 for those with dropout. The reduced range in the latter case is due to memory limitations encountered during simulation.

These results were obtained from single runs rather than averaged across multiple trials. As such, there may be some variability in the reported simulation times due to fluctuations inherent in the optimization process. However, the memory usage values provide a more stable and accurate reflection of resource requirements. This is because the simulations were executed using JAX with just-in-time (JIT) compilation, which compiles the full computation graph in advance. As a result, the memory footprint is primarily determined by circuit structure (e.g., number of qubits and layers) rather than by the number of optimization steps or epochs, and it exhibits the expected scaling with respect to circuit depth.

Accounting for the fact that each simulation was repeated 10 times to ensure statistical validity, and that some models were also used for the sample complexity study, we can estimate the total runtime of all simulations conducted in this study to be around 9,709 hours, approximately 404 days, and the total RAM used to be 15,947 GB.

**Quantum Architecture**	**Layers**									
	**1**	**2**	**3**	**4**	**5**	**6**	**7**	**8**	**9**	**10**
Angle Encoding	0.16	1.24	1.95	5.58	8.68	8.17	13.64	21.48	20.46	23.03
Angle Encoding + DR	0.50	1.74	3.87	8.42	9.44	12.95	21.99	25.31	39.44	42.34
Angle Encoding + Dropout	1.06	2.62	5.02	7.69	6.83	19.14	20.59	21.08	N/A	N/A
Angle Encoding + DR + Dropout	0.89	2.35	6.02	10.22	14.99	24.13	31.56	37.81	N/A	N/A
Amplitude Encoding	1.70	3.61	4.42	9.18	14.48	21.71	27.93	33.59	39.48	45.98
Amplitude Encoding + Dropout	1.44	3.18	7.00	9.64	14.49	16.96	18.00	22.35	N/A	N/A

TABLE I: Computational times in hours across varying layer depths for different quantum architectures. Values are rounded to two decimal places.

**Quantum Architecture**	**Layers**									
	**1**	**2**	**3**	**4**	**5**	**6**	**7**	**8**	**9**	**10**
Angle Encoding	2.08	4.47	8.00	12.45	17.96	24.49	32.05	40.66	50.26	60.83
Angle Encoding + DR	1.96	4.50	8.11	12.80	18.54	25.29	33.26	42.21	52.18	63.85
Angle Encoding + Dropout	1.93	4.44	7.91	11.88	17.11	23.29	30.59	38.89	N/A	N/A
Angle Encoding + DR + Dropout	2.04	4.80	8.70	13.30	19.61	26.85	35.45	45.31	N/A	N/A
Amplitude Encoding	3.21	6.82	11.91	18.45	26.47	35.97	46.88	59.29	73.26	88.54
Amplitude Encoding + Dropout	2.78	5.42	9.17	13.35	18.89	25.25	32.81	41.41	N/A	N/A

TABLE II: RAM requirements in Giga Bytes(GB) across varying layer depths for different quantum architectures. Values are rounded to 2 decimal places.

* francesco.ghisoni01@universitadipavia.it

II. DROPOUT STUDY

As an additional contribution to our investigation, we conducted a systematic study to determine the optimal dropout rate for our experiments. To do this, we selected the best-performing quantum neural network architecture from our initial analysis—Angle Encoding with Data Reuploading—and fixed the number of layers based on computational constraints (specifically, using 8 layers). We then applied five different dropout rates: 0.01, 0.02, 0.03, 0.04, and 0.05. For each dropout level, we trained the network 10 times to account for stochastic variation in the training process. The results of this analysis are presented in Figure 1, where it is evident that a dropout rate of 0.01 consistently yields the best performance. This value was therefore selected as the optimal dropout rate and used in all subsequent experiments involving dropout.

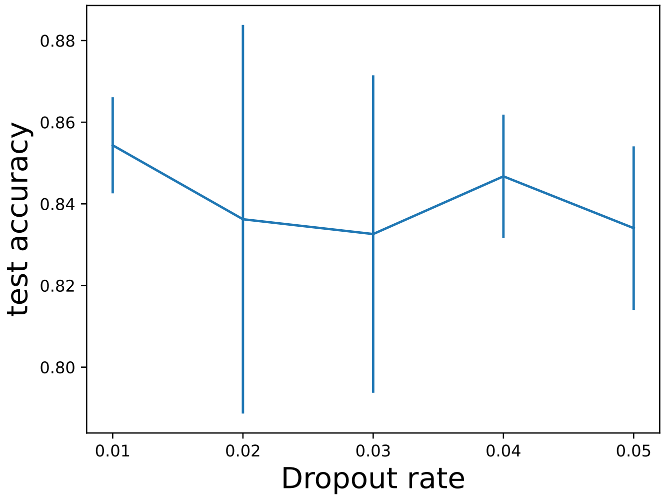

FIG. 1: Test accuracy of the QNN model (Angle Encoding with Data Reuploading, 8 layers) as a function of the dropout rate. The x-axis represents the applied dropout rate, while the y-axis shows the corresponding test accuracy averaged over 10 runs for each setting.

III. BARREN PLATEAU STUDY

While it is known that deep QNNs can, in principle, suffer from the barren plateau phenomenon—where gradients vanish exponentially with system size—we do not observe this issue in our study. One reason is that our circuit designs feature a linear scaling of trainable parameters with respect to the number of qubits, which has been shown to mitigate the onset of barren plateaus. Moreover, as discussed in the work by McClean et al. [1], for a system of 8 qubits, one would typically require circuits with approximately 100 layers of ansatz before encountering the barren plateau regime. Our circuits are considerably shallower and fall well below this threshold, with a maximum of 10 layers. To further reduce the risk of barren plateaus, we employed small-angle initialization, a known technique that helps preserve gradient magnitude in deeper circuits. To validate this empirically, we analyzed the gradients during training for the most performant QNN architecture in our study—9 layers of angle encoding with data reuploading—and observed that the gradient magnitude plateaued at a value of approximately 0.0167. The graph for these experiments can be seen in Figure 2. This indicates that gradients remain sufficiently large for effective optimization, particularly in our simulation setting, which is free from shot noise and device noise. We emphasize that this observation is limited to the noiseless simulation regime considered in this work; the presence of noise may induce or exacerbate barren plateaus even in shallow circuits, an effect that cannot be assessed here due to the computational infeasibility of large-scale noisy simulations.

IV. COMPARISON WITH XGBOOST

Gradient Boosted Decision Trees (GBDTs), and in particular algorithms such as XGBoost, LightGBM, and CatBoost, are widely regarded as state-of-the-art classical methods for structured tabular data. These models combine

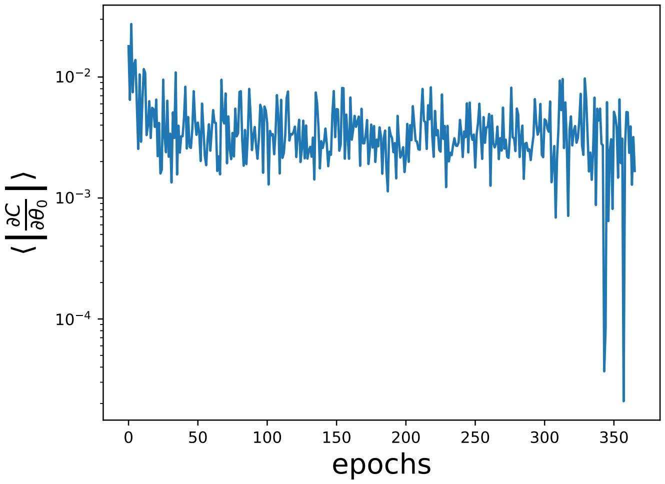

FIG. 2: Average gradient magnitude as a function of training epochs. The x-axis represents the number of training epochs, while the y-axis shows the average gradient of the cost function. This plot illustrates how the gradient evolves during training and highlights the onset of gradient plateauing.

ensembles of decision trees trained sequentially via gradient boosting and are known for their strong performance across a wide range of tabular datasets, including many benchmarks in medical and clinical data analysis. As such, they often represent a highly competitive classical baseline, particularly when feature engineering and hyperparameter tuning are carefully performed.

In the main text of this manuscript, we intentionally focus on a direct comparison between NNs and QNNs. This choice is motivated by the desire to compare models that share a similar functional form and learning paradigm, namely parameterized, gradient-based models trained via backpropagation. Such a comparison allows for a clearer assessment of how quantum circuit–based models relate to their classical neural counterparts in terms of expressivity, optimization behavior, and sample complexity, without introducing confounding differences arising from fundamentally distinct algorithmic principles.

Nevertheless, we acknowledge that neural networks are not always the strongest classical baseline for structured tabular data, and that ensemble methods such as XGBoost often outperform feed-forward NNs in practice. For this reason, we include in this Supplementary Material an additional comparison with XGBoost to provide broader classical context for the results presented in the main manuscript. This supplementary analysis is not intended to replace the NN–QNN comparison, but rather to situate the observed performance of QNNs within the landscape of strong, non-neural classical methods commonly used in applied machine learning.

By reporting XGBoost results alongside those of NNs and QNNs, we aim to offer a more complete perspective on the relative strengths and limitations of quantum neural networks, while preserving the conceptual clarity of the main text, which focuses on neural architectures across classical and quantum settings. The results for this comparison can be seen in table III.

To ensure a fair and statistically consistent comparison with the models analyzed in the main text, the XGBoost classifier was trained and evaluated following the same protocol adopted for classical and quantum neural networks. In particular, the model was trained and tested 10 independent times using different random seeds, and performance was reported as the mean test accuracy with the corresponding uncertainty. Using this procedure, XGBoost achieved a final test accuracy of 85.43±0.004%. While this performance is competitive, it does not exceed that of the best-performing QNNs based on angle encoding, nor that of the best classical neural network. Instead, the XGBoost model outperforms only the QNNs employing amplitude encoding, which consistently achieved lower test accuracies in the main analysis. This result highlights that, within the scope of this dataset and experimental setup, strong ensemble-based classical methods do not uniformly dominate neural architectures, and that QNNs with appropriate encoding choices remain competitive even when compared against widely used state-of-the-art models for tabular data.

The sample complexity analysis including XGBoost was conducted using the exact same experimental setting adopted in the main manuscript with results that can be seen in figure 3. In particular, the training dataset was subsampled to fractions of 10%, 30%, 50%, 70%, and 100% of the original training set, and for each subset size the models were trained and evaluated 10 independent times using different random seeds and data splits. Performance is reported in terms of mean test accuracy with the corresponding uncertainty, ensuring full consistency with the

Architecture	Best Test Accuracy
QNN - Amplitude Encoding	82.86 ± 0.01%
QNN - Amplitude Encoding + Dropout	80.43± 0.01%
QNN - Angle Encoding	86.67 ± 0.01%
QNN - Angle Encoding + Data Re-uploading	86.84 ± 0.01 %
**QNN - Angle Encoding + Dropout**	**87.46 ± 0.01%**
QNN - Angle Encoding + Data Re-uploading + Dropout	86.09 ± 0.01%
Classical Neural Network(52-52-52)	86.67± 0.07%
**XGBoost**	**85.43± 0.004%**

TABLE III: This table provides a summarization of the results from our large-scale statistical analysis and XGBoost.

methodology used for classical and quantum neural networks.

The results show that XGBoost exhibits strong performance in the low-to-intermediate data regimes, but with a behavior that differs from both classical and quantum neural networks. For the smallest training set size (10%), XGBoost ranks as the third best-performing model, outperforming several neural architectures but remaining inferior to the best-performing shallow QNNs in this regime. As the training set size increases to 30% and 50%, XGBoost becomes the best-performing model overall.

However, as the training set size is further increased, the performance of XGBoost plateaus, while both classical and quantum neural networks with higher expressive capacity continue to improve. In the larger data regimes (70% and 100% of the training set), more complex classical neural networks and deeper QNNs overtake XGBoost in test accuracy.

Overall, this supplementary analysis confirms that XGBoost constitutes a strong and relevant classical baseline, particularly in intermediate data regimes, while also reinforcing the main conclusions of the paper: shallow QNNs can be competitive in data-scarce settings, and more expressive neural models—both classical and quantum—can surpass tree-based ensembles as data availability increases.

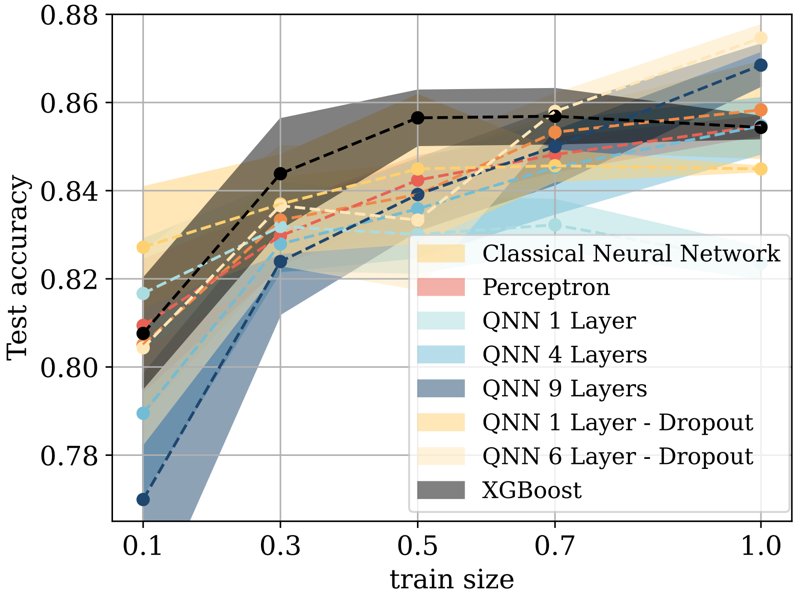

FIG. 3: Results of the sample complexity study including XGBoost.

[1] J. R. McClean, S. Boixo, V. N. Smelyanskiy, R. Babbush, and H. Neven, Barren plateaus in quantum neural network training landscapes, Nature Communications **9**, 4812 (2018).
